# NAP1L1 promotes tumor proliferation through HDGF/C-JUN signaling in ovarian cancer

**DOI:** 10.1186/s12885-022-09356-z

**Published:** 2022-03-29

**Authors:** Xiaohua Zhu, YingYing Xie, Wenyan Huang, Zigui Chen, SuiQun Guo

**Affiliations:** 1 Department of Obstetrics and Gynecology, The Third Affliated Hospital, Southern Medical University, Guangzhou, 510500 Guangdong China; 2 The Second School of Clinical Medicine, Zhujiang Hospital, Southern Medical University, Southern Medical University, Guangzhou, Guangdong, 510280 China; 3 Cancer Center, Integrated Hospital of Traditional Chinese Medicine, Southern Medical University, Guangzhou, 510315 Guangdong China; 4 First People’s Hospital of Zhaoqing, Zhaoqing, 2102013 Guangdong China; 5 Department of Obstetrics and Gynecology, The Third Affliated Hospital, Southern Medical University, 183 Zhongshan Dadaoxi West, Guangzhou, 510500 Guangdong China

**Keywords:** NAP1L1, HDGF, C-JUN, CCND1, Ovarian cancer, Proliferation

## Abstract

**Background:**

Nucleosome assembly protein 1-like 1 (NAP1L1) is highly expressed in various types of cancer and plays an important role in carcinogenesis, but its specific role in tumor development and progression remains largely unknown. In this study, we suggest the potential of NAP1L1 as a prognostic biomarker and therapeutic target for the treatment of ovarian cancer (OC).

**Methods:**

In our study, a tissue microarray (TMA) slide containing specimens from 149 patients with OC and 11 normal ovarian tissues underwent immunohistochemistry (IHC) to analyze the correlation between NAP1L1 expression and clinicopathological features. Loss-of- function experiments were performed by transfecting siRNA and following lentiviral gene transduction into SKOV3 and OVCAR3 cells. Cell proliferation and the cell cycle were assessed by the Cell Counting Kit-8, EDU assay, flow cytometry, colony formation assay, and Western blot analysis. In addition, co-immunoprecipitation (Co-IP) and immunofluorescence assays were performed to confirm the relationship between NAP1L1 and its potential targets in SKOV3/OVCAR3 cells.

**Results:**

High expression of NAP1L1 was closely related to poor clinical outcomes in OC patients. After knocking down NAP1L1 by siRNA or shRNA, both SKOV3 and OVCAR3 cells showed inhibition of cell proliferation, blocking of the G1/S phase, and increased apoptosis in vitro. Mechanism analysis indicated that NAP1L1 interacted with hepatoma-derived growth factor (HDGF) and they were co-localized in the cytoplasm. Furthermore, HDGF can interact with jun proto-oncogene (C-JUN), an oncogenic transformation factor that induces the expression of cyclin D1 (CCND1). Overexpressed HDGF in NAP1L1 knockdown OC cells not only increased the expression of C-JUN and CCND1, but it also reversed the suppressive effects of si-NAP1L1 on cell proliferation.

**Conclusions:**

Our data demonstrated that NAP1L1 could act as a prognostic biomarker in OC and can interact with HDGF to mediate the proliferation of OC, and this process of triggered proliferation may contribute to the activation of HDGF/C-JUN signaling in OC cells.

**Supplementary Information:**

The online version contains supplementary material available at 10.1186/s12885-022-09356-z.

## Background

Ovarian cancer (OC) is the most deadly gynecological tumor in female reproductive organs in China [1], and the underlying molecular mechanisms in OC development remain unclear. It is difficult to diagnose OC at an early stage, and the treatment options following diagnosis are limited. Therefore, there is an urgent need to identify innovative biological targets.

Nucleosome assembly protein 1-like 1 (NAP1L1) is a member of the nucleosome assembly protein l-like family that includes NAP1L1, NAP1L2, NAP1L3, NAP1L4, and NAP1L5 [1], which was first detected in human thymus tissue [2]. The functions attributed to NAP1L1 proteins include nucleosome assembly, histone transport, histone eviction, transcriptional regulation, and cell cycle progression [3]. Among all members of the nucleosome assembly protein 1-like family, NAP1L1 has a higher nucleosome assembly capability [4]. NAP1L1 has been reported to be highly expressed in several types of human malignancies, including colorectal cancer, hepatocellular cancer, lung adenocarcinoma, and renal cancer [5–8]; however, its expression level in OC is not yet clear. The purpose of this study is to investigate the expression of NAP1L1 in OC and to analyze its prognostic significance.

We found that high expression of NAP1L1 was associated with poor prognosis in OC patients. The knockdown of NAP1L1 inhibited cell proliferation, blocked the G1/S transition, and induced apoptosis in OC. Further, Mechanism analysis indicated that NAP1L1 can interacting with HDGF, which recruits C-JUN and thus promotes cell cycle signal transition, this ultimately promotes OC proliferation by increasing the expression of C-JUN and CCND1. Accordingly, targeting NAP1L1 may be an alternative strategy for the treatment of OC.

## Methods

### Cells and patients

Cell lines SKOV3 and OVCAR3 used in this study were obtained from the Shanghai Institute of Cell Biology, Chinese Academy of Sciences (Shanghai, China). Cells were cultured in RPMI-1640 medium (ChongQing, China) with 20% fetal bovine serum (Nobimpex, Germany) at 37 °C with 5% CO2.

### Transfection

We used lentiviral mediated shRNA (Supplementary Table S1) interference to achieve stable NAP1L1 knockdown OC cells. Lentiviral NAP1L1 shRNA (shNAP1L1) and negative control (shNC) were generated according to the manufacturer’s protocol (RiboBio Guangzhou, China). Small interfering siRNAs (Supplementary Table S1) were designed by RiboBio Corporation (Guangzhou, China). Plasmids were obtained from Vigene Biosciences Corporation (Shandong, China), and the transfection of plasmids or siRNA was performed by Lipofectamine® 3000(Invitrogen; Thermo Fisher Scientific, USA) according to the manufacturer’s protocol.

### Cell Counting Kit-8 (CCK-8) assay

In brief, 2 × 10^3^ cells were plated in 96-well plates after being incubated for 1, 2, 3, and 4 days. At the same time point, 10% CCK-8 (APExBIO, USA) was added in a 37 °C humidified incubator. Two hours later, the absorbance value (OD) was measured at 450 nm with a microplate reader (Thermo Scientific Multiskan Sky, USA).

### Colony formation assay

Cells at a density of 200 cells/well were plated in six-well plates. After culture for 14 days, the colonies generated were fixed with methanol (DAMAO, TJ, China) for 15 min, and then they were stained with crystal violet staining solution (LEAGENE, BJ, China) for 15 min. Next, they were photographed under an optical microscope to count the number of colonies (> 50 cells).

### Quantitative reverse transcription polymerase chain reaction (qRT-PCR)

Total RNA was extracted from SKOV3 and OVCAR3 cells, and cDNA was generated by using the Evo M-MLV RT-PCR Kit (AG11601/AG11602, Hunan, China). SYBR® Green Master Mix was used for qPCR. The primers are presented in Supplementary Table S2. The Bio-Rad CFX 96 and Bio-Rad T100 detection systems were used for QPCR and RT-qPCR.

### Cell cycle assay

The cells for cell cycle were in the logarithmic phase, and after transfection with siNAP1L1 for 24 h, they were harvested and stained with 70% ethanol at 4℃ overnight following one application of phosphate buffered saline (PBS) wash. Flow cytometry was performed the next day according to the manufacturer's instructions, Cell Cycle and Apoptosis Analysis Kit (Leagene Biotechnology, BJ, China).

### Cell apoptosis assay

Cell apoptosis was detected using a BD Annexin V-FITC apoptosis assay kit (BD Biosciences Pharmingen, San Diego, US) according to the manufacturer's instructions. Flow cytometry (BD FACSCalibur, USA) was performed to assess cell apoptosis.

### Immunofluorescence and confocal microscopy

Cells were separated and seeded on a 35 mm glass bottom cell culture dish (SORFA, ZJ, China) at a density of 5000 cells/well. After cell adherence, the cells were fixed with paraformaldehyde (4%) and permeabilized with Triton X-100 (0.2%). The cells were then incubated with specific antibodies (The antibodies are presented in Supplementary Table S3), counterstained with DAPI (0.2 mg/ml), and imaged using a Carl Zeiss LSM800 confocal laser scanning microscope.

### Co-immunoprecipitation (Co-IP)

The Pierce ™ Co-IP kit (Thermo Fisher Scientific, Sweden, USA) was used according to the manufacturer's instructions. Specific antibodies (10 μg) or IgG were added to 5 mg protein for overnight incubation at 4℃. Beads were washed, and the result was filmed using the Minichemi ™ Chemiluminescence Imaging System (Sagecreation, BJ, China).

### Western blotting

Western blotting was conducted as previously described [9]. The antibodies (Supplementary Table S3) used were anti-GAPDH(pAb AP0063), NAP1L1(mAb ab178687), hepatoma-derived growth factor (HDGF)(pAb 11,344–1-AP), jun proto-oncogene (c-JUN)(mAb #9165), and CCND1(mAb#6086–1-Ig).

### Immunohistochemistry staining (IHC)

To determine the expression levels of NAP1L1 in ovarian cancer, ovarian cancer tissue microarrays (TMA) (HOvaC151Su01) including malignant human ovarian tissues (*n* = 149) was provided by Outdo Biotech (Shanghai, China), the TMA including different histologic types of epithelial ovarian cancer (EOC)(*n* = 149), including serous adenocarcinoma, mucinous adenocarcinoma, endometrioid adenocarcinoma, and clear-cell adenocarcinoma, so all the samples are EOC subtype. 11 cases of normal ovarian tissue were collected from Zhujiang Hospital, Southern Medical University/The Second School of Clinical Medicine, Southern Medical University. IHC was performed according to the standard protocols. The percentage of positive cancer cells was assigned to one of four rankings [10]. A staining index > 6 was classified as high expression, and a staining index ≤ 5 was classified as low expression. Staining index performed blinded by an independent technician.

### Statistical analysis

The chi-square test, Kaplan Meier and Cox regression was used to analyze the relationship between NAP1L1 expression and clinicopathological characteristics. Survival curves were plotted using the Kaplan–Meier method and compared by log-rank test, *p* < 0.05 was considered significant. The data are presented as the mean ± SD of at least three independent experiments. The difference between two groups was analyzed by using the Student’s t-test. Statistical analysis was conducted using Prism GraphPad Software. Throughout the text, the following symbols are used to denote statistical significance: *p < 0.05, **p < 0.01, and ***p < 0.001.

## Results

### High expression of NAP1L1 is associated with poor clinical outcomes

**Fig. 1:**
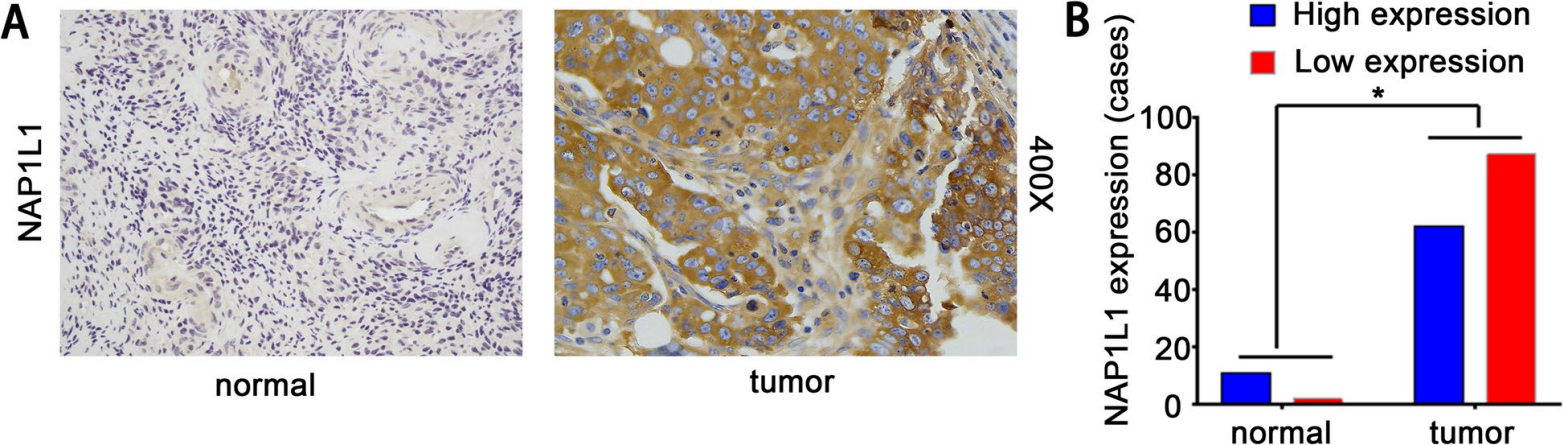
NAP1L1 overexpression in serous ovarian cancer tissues with IHC. **A**-**B** NAP1L1 overexpression in serous ovarian cancer tissues with IHC, NAP1L1 expression in normal context and serous ovarian cancer tissues (*P* < 0.012). *p < 0.05, **p < 0.01, and ***p < 0.001

**Fig.2:**
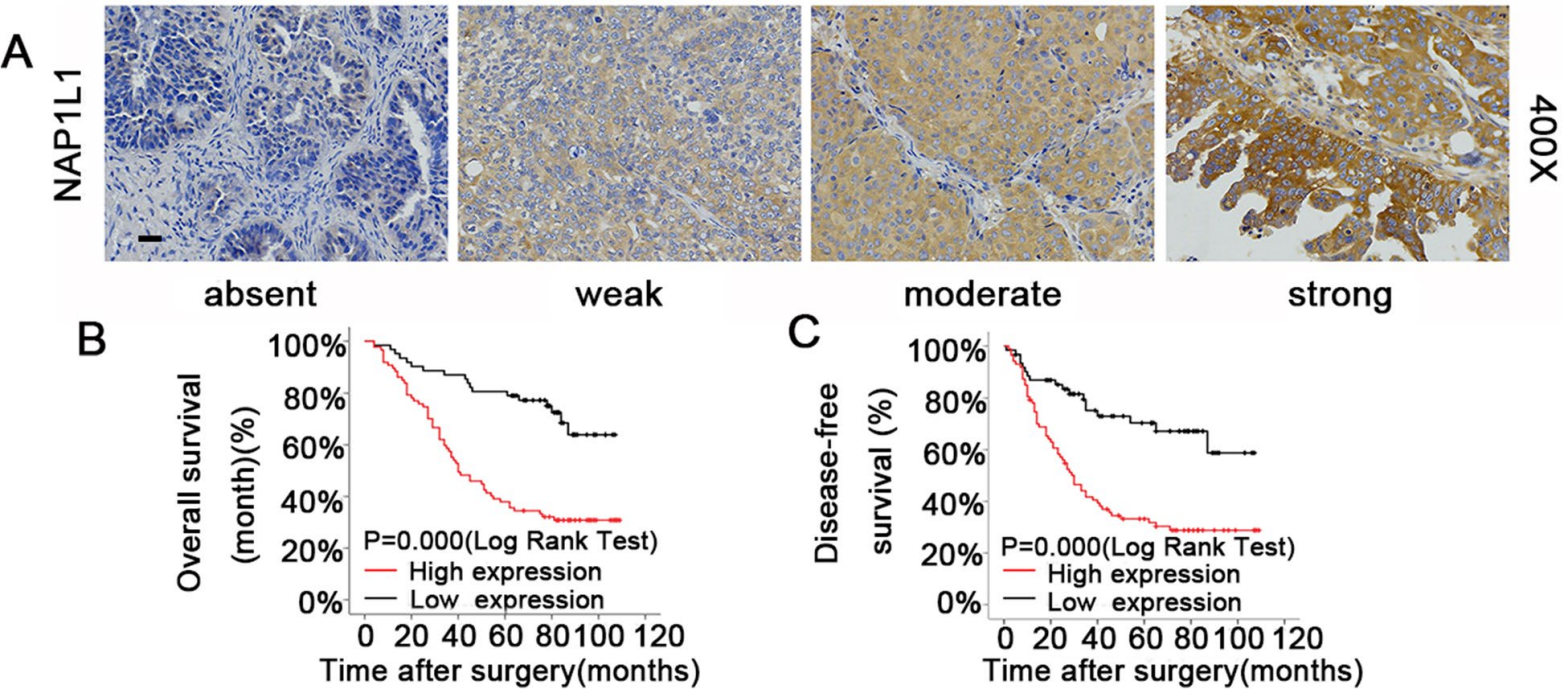
NAP1L1 is upregulated in OC and is associated with poor clinical prognosis. **A** Different IHC staining patterns of NAP1L1 expression in OC samples (Scale bar, 200 mm). **B**-**C** Kaplan–Meier survival analysis of OC patients with high or low expression of NAP1L1. Log-rank test was used to calculate the disease-free survival and overall survival rates

Ovarian cancers are grouped into three categories based on the cell type of origin: epithelial, stromal, and germ cell cancer. Among them, epithelial ovarian cancer (EOC) accounts for 90–95% of ovarian malignancies. EOC is further grouped into five histological subtypes: high-grade serous carcinomas (HGSC, 70%-74%), endometrioid carcinomas (EC, 7–24%), clear cell carcinomas (CCC, 10%-26%), low-grade serous carcinomas (LGSC, 3%-5%), and mucinous carcinomas (MC, 2%-6%) [11]. The tissue array (HOvaC151Su01) included 149 cases of epithelial ovarian cancer (EOC), The TMA including different histologic types of epithelial ovarian cancer, including serous adenocarcinoma, mucinous adenocarcinoma, endometrioid adenocarcinoma and clear-cell adenocarcinoma, so all the samples are EOC subtype. To determine the expression levels of NAP1L1 in ovarian cancer, 149 ovarian cancer tissues and 11 normal ovarian epithelial tissues were collected. NAP1L1 expression levels were further examined in these tissues by immunohistochemistry (IHC). In ovarian cancer tissues group, the results (Figs. 1A and B) showed that 62/149 patients (41.61%) ha asd low expression and 87/149 patients (58.38%) had high expression; however, among patients from whom 11 normal ovarian epithelial tissues stained with NAP1L1 were obtained, 9/11 (81.81%) had low expression and 2/11 (18.18%) had high expression, and there was significant difference between the tumor and normal groups (P < 0.012). We found that NAP1L1 expression staining was located within the cytoplasm and NAP1L1 overexpression was detected in epithelial ovarian cancer tissues with IHC. To explore the correlation between NAP1L1 expression and survival in OC patients, we analyzed tumor tissues obtained from 149 patients with ovarian cancer using IHC. NAP1L1 expression was classified into the high expression and low expression groups, as shown by staining (Fig. 2A). The basic clinicopathological characteristics are listed in Table 1, The results showed that 87 out of 149 (58.38%) patients had high expression and 62 out of 149 (41.61%) patients had low expression. In addition, Kaplan–Meier survival analysis demonstrated that OC patients with high NAP1L1 expression had worse disease-free survival and overall survival rates than OC patients with low NAP1L1 expression (Fig. 2B and 2C). Multivariate Cox regression analysis showed that NAP1L1 expression was an independent prognostic factor for OC (Table 2). These results suggest that NAP1L1 is a potential prognostic factor and it acts as an oncogene in the progression of OC.

**Table1:** Correlation of NAP1L1 protein expression with clinicalpathological parameters

Parameters	Total	Low expression	High expression	*P*-value
Age(y)				
≤ 51	75	35	40	
> 51	74	27	47	0.207
Pathological Grading				
I/II	22	13	9	
III	99	31	68	0.014
T stage				
T1/T2	44	26	18	
T3	105	36	69	0.005
N stage				
N0	111	57	54	
N1	38	5	33	0.000
M stage				
M0	117	57	60	
M1	32	5	27	0.001
Clinical stages				
I/II	44	26	18	
III/IV	105	36	69	0.005
Vital status				
Alive	71	44	27	
Dead	78	18	60	0.000
Tumor recurrence				
No	29	17	12	
Yes	120	45	75	0.038

**Table2:** Multivariate Analysis of Risk Factors for Overall Survival

Characteristics	Hazard Ratio	95%Confidence Interval	*P*-value
Age(y)			
≤ 51	1		
> 51	0.731	0.452 to 1.181	0.201
Pathological Gradig			
I/II	1		
III	1.732	0.086 to 3.484	0.124
T stage			
T1/T2	1		
T3	7.553	2.679 to 21.294	0.000
N stage			
N0	1		
N1	1.591	0.801 to 3.162	0.185
M stage			
M0	1		
M1	2.575	1.305 to 5.081	0.006
Clinical stages			
I/II	1		
III/IV	11.814	4.312 to 32.365	0.000
NAP1L1 Status			
Low expression	1		
High expression	2.045	1.145 to 3.657	0.016

### Silencing NAP1L1 suppresses the proliferation of OC cells

**Fig. 3:**
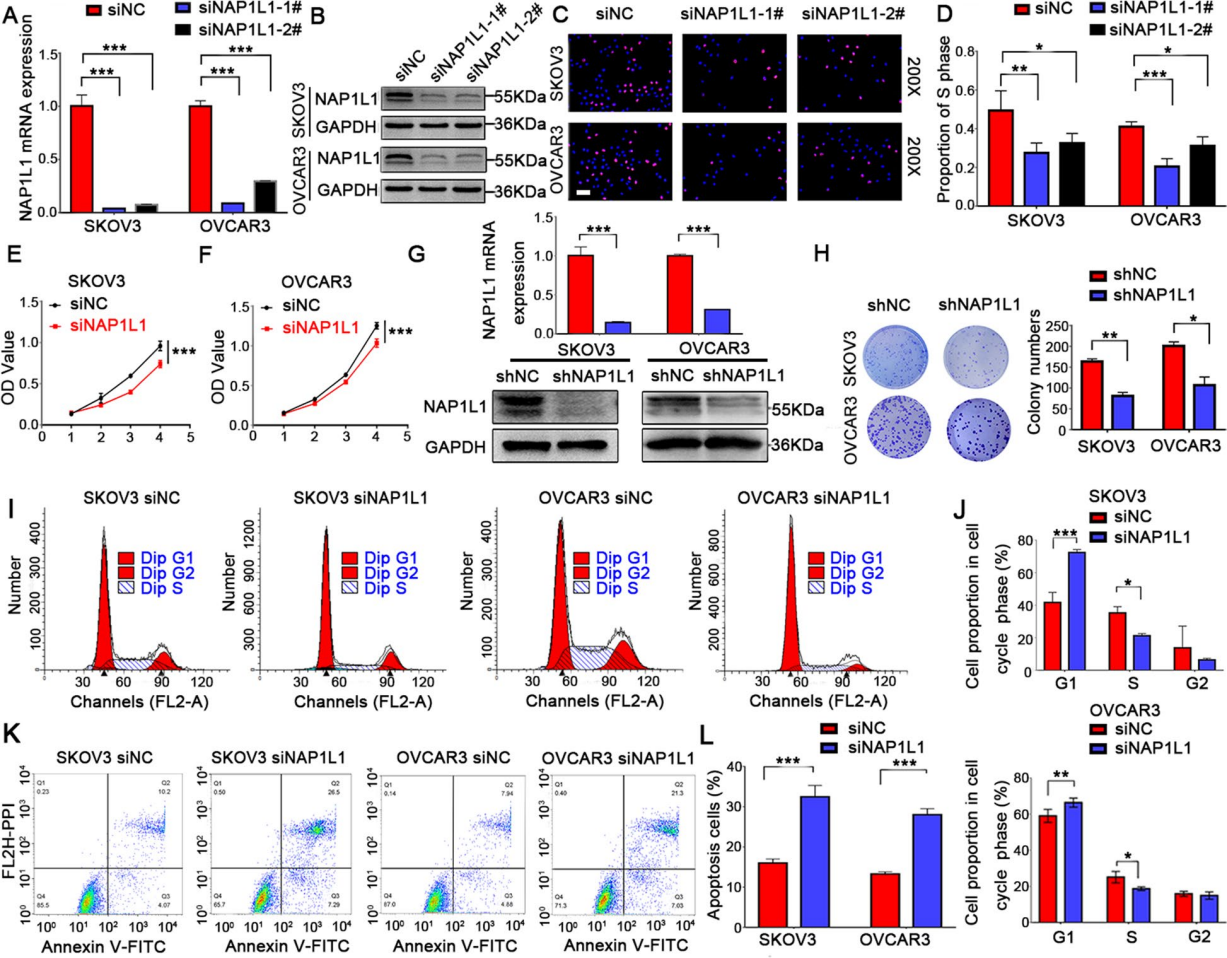
NAP1L1 suppressed the proliferation of OC cells (instantaneous and stable interference). **A**-**B** The expression of NAP1L1 in OC cell lines was examined by RT-qPCR and Western blotting to verify the efficiency of interference following instantaneous infection of siNAP1L1 and (G) lentiviral infection of shNAP1L1. **C**-**D** EdU assays (scale bar, 50 μm) were conducted after transfection with siNAP1L1#1 or #2. The first segment was selected for subsequent experiments due to the higher efficiency. **E**–**F** MTT assays were conducted after transfection with siNAP1L1#1. **H** Colony numbers of shNAP1L1 and shNC. **I**-**J** Cell cycle distribution of siNAP1L1/siNC in OC cells and statistical analysis of cell percentages in G1, S, G2 in the above cells. **K**-**L** The apoptosis of the above cells was analyzed by flow cytometry. *p < 0.05, **p < 0.01, and ***p < 0.001

We applied lentiviral mediated shRNA to knock down the NAP1L1 expression in OC cell lines, including SKOV3 and OVCAR3. First, 2 siRNAs targeting NAP1L1 (siNAP1L1) were tested by qRT-PCR and Western blot analysis (Fig. 3A and B). The EdU assays revealed that knockdown of NAP1L1 significantly suppressed DNA synthesis in OC cells (Fig. 3C and D). Due to enhanced inhibitory efficiency, we chose siNAP1L1-1# for the subsequent experiments. To further explore the role and mechanism of NAP1L1 in tumorigenesis, lentiviral mediated shRNA interference was used to achieve stable NAP1L1 knockdown cells (Fig. 3G). After knocking down NAP1L1, both SKOV3 and OVCAR3 cells showed a significant decrease in cell numbers (*p* < 0.001) (Fig. 3E and F) and colony formation (Fig. 3H). These results indicated that silencing NAP1L1 significantly inhibited cell proliferation in OC. To test whether inhibition of cell proliferation was a result of cell cycle arrest, we next examined the cell cycle distribution of siNAP1L1/siNC in OC cells. As shown in (Fig. 3I and J), NAP1L1 knockdown cells exhibited a higher percentage of cells in the G1 phase and a decrease in the cell cycle progression to the S phase. In order to demonstrate whether cell cycle arrest contributed to downregulation of NAP1L1 and thus enhanced cell death, we further assessed cell apoptosis. Silencing of NAP1L1 yielded a significant increase in the apoptotic cell population (Fig. 3K-L). These results indicate that NAP1L1 enhances cell growth, at least partially, by inducing G1/S transition.

### NAP1L1 interacts with HDGF and HDGF interacts with C-JUN

**Fig. 4:**
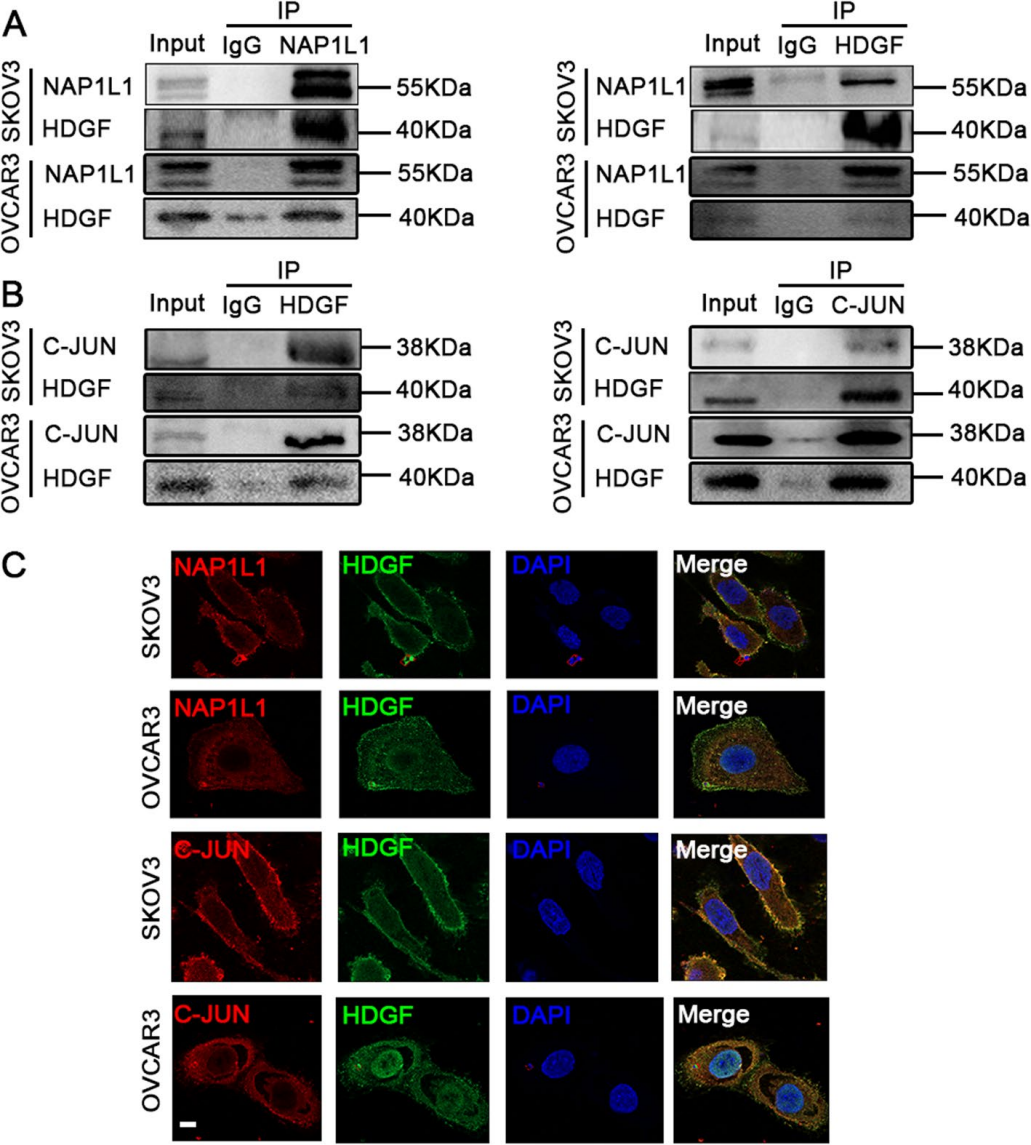
NAP1L1 protein interacts with HDGF and HDGF interacts with C-JUN. **A** Co-IP experiments detected the interaction of exogenous NAP1L1 and HDGF in both SKOV3 and OVCAR3 cells. **B** Co-IP experiments detected the interaction of HDGF and C-JUN in OC cells. **C** NAP1L1 and HDGF co-localized in the cell cytoplasm as observed by immunofluorescent staining. HDGF and C-JUN protein co-localization was observed in the cytoplasm and nucleus in both cell lines (Scale bar, 5 μm)

To further explore the role of NAP1L1 in OC, we focused on the protein interacting with NAP1L1. Mass spectrometry was used to predict the potential target in our preliminary work (unpublished data). We immunoprecipitated the NAP1L1 protein with an anti-NAP1L1 antibody and identified the proteins that may directly interact with NAP1L1. HDGF has been newly predicted as a candidate interacting factor. HDGF has been reported to drive the progression of OC [12]. To validate the protein interaction between NAP1L1 and HDGF, a Co-IP assay was carried out, as shown in Fig. (4A). As expected, in a reciprocal Co-IP with HDGF, NAP1L1 was detected in the immunoprecipitated complex. Moreover, NAP1L1 and HDGF were co-localized in the cell cytoplasm of both cell lines, as observed by immunofluorescence staining (Fig. 4C). These results indicate the physical interaction between NAP1L1 and HDGF. Elucidation of potential downstream interactions with NAP1L1 was performed by employing the BIOGRID and STRING databases. The single protein function partner network of HDGF analysis indicated that the downstream factor C-JUN may also interact with HDGF (Supplementary Figs. 1A and B). The transcription factor C-JUN is a downstream driver of the Wnt/β-catenin signaling pathway [13]. Previous studies have shown Wnt/ β-catenin and C-JUN as positive downstream signaling pathways of MYH9, playing an important role in the development of HCC [9]. The interaction between HDGF and C-JUN was detected by the endogenous Co-IP assay (Fig. 4B). The results demonstrated that HDGF interacted with C-JUN in OC cells. In addition, confocal microscopy was used to observe the subcellular localization of HDGF and C-JUN following immunostaining (Fig. 4C), and the result indicates that HDGF and C-JUN are co-localized in the cell cytoplasm and nucleus.

### Inhibition of cell proliferation by siNAP1L1 is reversed by HDGF overexpression in OC cells

**Fig. 5:**
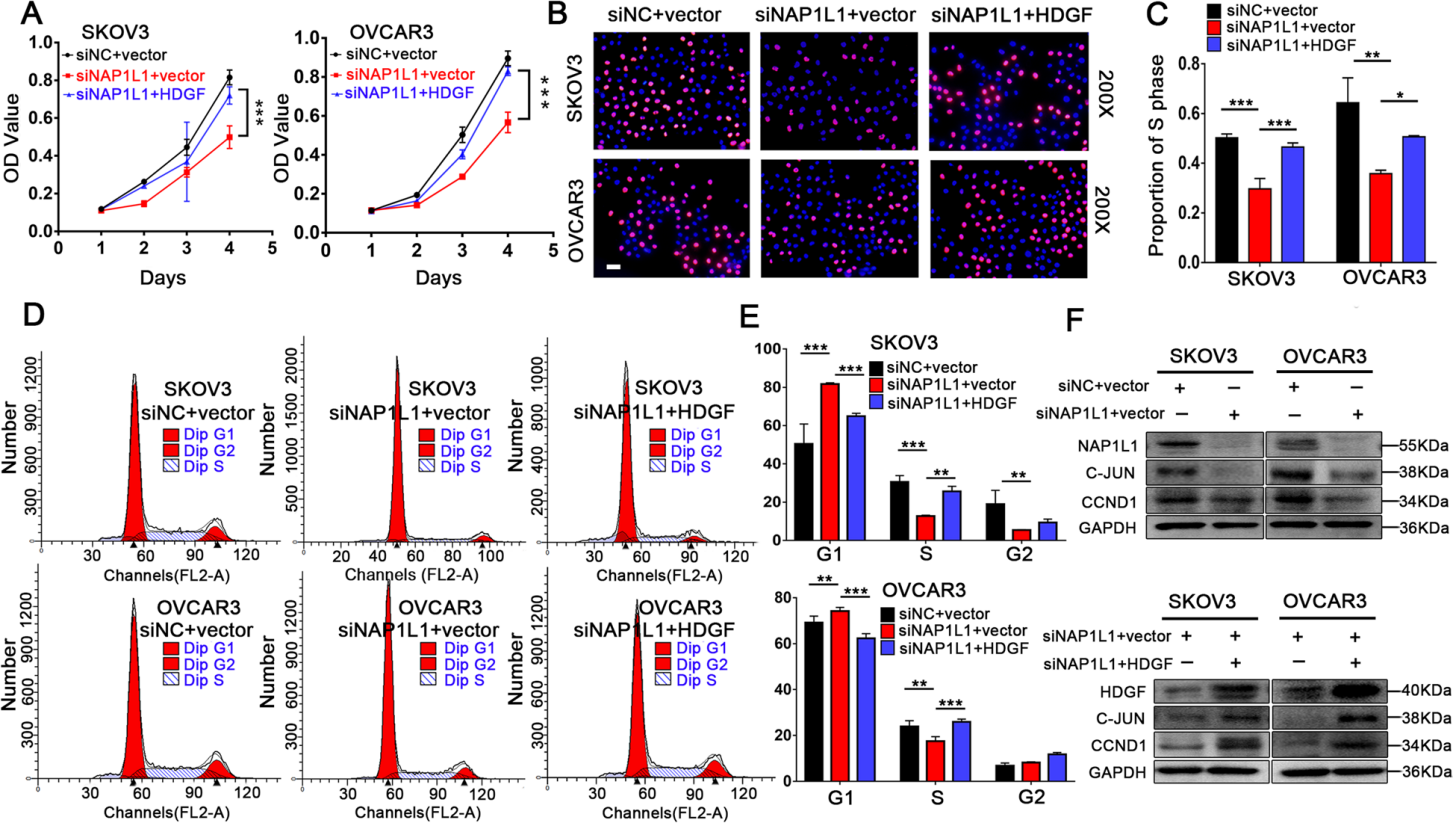
Transfecting HDGF reverses the suppressive effects in OC cells. **A** Transfecting HDGF reverses cell proliferation identified using CCK-8 assay in OC cells. **B**-**C** Transfecting HDGF reverses cell proliferation identified using EdU assay (scale bar, 50 μm). **D**-**E** Flow cytometric analysis indicates transfecting HDGF reverses cell cycle distribution. **F** The efficiency of the transiently transfected HDGF plasmid into the NAP1L1 knock down cells was examined by Western blotting. In those NAP1L1 silenced cells, we observed a decrease in the expression of C-JUN and CCND1, with the overexpressing of HDGF, the C-Jun and CCND1 protein expression also increased. *p < 0.05, **p < 0.01, and ***p < 0.001

CCK-8 assays (Fig. 5A) and EdU assays (Fig. 5 B and C) were performed to demonstrate whether HDGF reverses siNAP1L1 inhibited OC cell proliferation, and both assays verified that overexpression of HDGF reversed the proliferation of NAP1L1 silenced OC cells. In addition, cell cycle assays were performed to explore the reverse effects of overexpressed HDGF on siNAP1L1 inhibited OC cells entering into the G1/S phase (Fig. 5D and E). Western blotting was performed to explore the underlying mechanism (Fig. 5F). Notably, NAP1L1 expression was downregulated in NAP1L1 suppressed OC cells, based on the NAP1L1 silencing induced G1/S blocking. We next tested the expression of relevant downstream cell cycle proteins C-JUN and CCND1 in OC cells. In the NAP1L1 silenced cells, we observed a decrease in the expression of C-JUN and CCND1. Overexpression of HDGF reversed the siNAP1L1 mediated inhibition of C-JUN and CCND1, which suggests that the overexpression of HDGF can enhance binding with C-JUN and thus induce upregulation of the expression of the downstream proteins C-JUN and CCND1.

## Discussion

In previous studies, NAP1L1 has been shown to promote tumor cell proliferation in various human malignancies, including colorectal cancer, hepatocellular cancer, lung adenocarcinoma, renal cancer, and neuroendocrine neoplasms [5–8, 14], and overexpression of NAP1L1 is related to a poor prognosis.

In The Kaplan–Meier Plotter(The KM plotter) data(https://kmplot.com/analysis/index.php?p=service), The KM plotter is capable to assess the effect of any gene or gene combination on survival in breast, ovarian, lung, gastric, colon, prostate, GBM, LGG, melanoma, DLBCL, RCC, AML, and 14 other tumor types using over 50,000 samples measured using gene arrays, RNA-seq or next generation sequencing (for mutation data). In The KM plotter data (Supplementary Figures. 2): high expression of NAP1L1 was closely related to poor clinical outcomes in ovarian cancer patients(*p* = 0.013). In this study, we also found that the Kaplan Meier plotter analysis showed that the NAP1L1 high expression group tended to have poor disease-free survival and overall survival rates; additionally, OC patients with advanced-stage disease had a higher NAP1L1 expression. This upregulation of NAP1L1 was closely associated with a poor prognosis in OC patients; therefore, consistent with previous research on other malignancies, our findings indicate that NAP1L1 might be a biomarker of poor prognosis in OC.

As an important member of the nucleosome assembly protein l-like family, NAP1L1 has been shown to regulate DNA replication and chromatin formation, which contribute to various processes [15]. A study has revealed that NAP1L1 is highly conserved compared with NAP1L2, L3, and L5 [16], suggesting a fundamental significance of NAP1L1 in cellular functions. In pancreatic neuroendocrine neoplasms, NAP1L1 has been proved to be involved in promotion of tumor cell proliferation and regulation of cell entry into the S phase [14]. In murine induced pluripotent stem cells, NAP1L1 has been proved to be involved in cell proliferation and G2/M transition[17]. Consistent with the above studies, in a subsequent investigation, we observed that knockdown of NAP1L1 by siRNA or shRNA significantly decreased cell proliferation and blocked cells in the G1 and S phases in SKOV3/OVCAR3 cells. Due to G1 phase and S phase block, cell proliferation and apoptosis status are subject to change [18]. Previous studies have shown that NAP1L1 downregulation renders the cell vulnerable to apoptotic cell death [19]. Similarly, in our study, we observed a significant increase in apoptosis in NAP1L1 downregulated OC cells.

In lung adenocarcinoma (AC) derived A549 cells, NAP1L1 regulates the NF-κB signaling pathway by modifying gene expression of the anti-apoptotic factor Mc1-1[19]. NAP1L1 may contribute to the aggressive nature of tumor cells through the PI3K/AKT/mTOR signaling pathway in lung AC [20]. In pancreatic neuroendocrine neoplasms, NAP1L1 promotes tumor cell proliferation and regulates cell entry into the S phase via inhibition of the mTOR pathway [14]. However, the molecular regulatory mechanism of NAP1L1 in OC remains unclear. To explore the mechanism of NAP1L1 in OC, we focused on the protein interacting with NAP1L1. Prior investigation performed by our group using mass spectrometry (unpublished data) had determined that NAP1L1 was a potential candidate interaction protein of HDGF in endometrial carcinoma. HDGF, which belongs to an eponymous family of proteins, is a heparin binding growth factor originally identified from the conditioned medium of human liver cancer cell line Huh-7 [21]. HDGF overexpression has been reported in many types of cancers [22–24]. HDGF has also been reported to be implicated in many cancer processes, including cancer growth, apoptosis, angiogenesis, and metastasis [25]. Studies have also found that HDGF expression correlates with an unfavorable prognosis and it can be considered as an independent prognostic factor in OC patients [26]. These findings indicate the significance of HDGF in OC development. To further validate the protein interaction between NAP1L1 and HDGF in our study, endogenous Co-IP was applied, and it was found that NAP1L1 and HDGF were co-localized in cell cytoplasm in OC cells. These results indicate the physical interaction between NAP1L1 and HDGF. We have knockdown NAP1L1 in SKOV3 and OVCAR3 cells and examine the NAP1L1/HDGF expression, results are as follows (Supplementary Figures. 3). Knocking down NAP1L1 reduced HDGF expression, after the overexpression of NAP1L1 in SKOV3 and OVCAR3 cells, western blotting demonstrated that the protein level of HDGF is increased, which demonstrated that HDGF is the downstream factor of NAP1L1.

To clarify the potential downstream molecular mechanism of NAP1L1 for promoting cell proliferation and cell cycle transition via HDGF in OC cells, the BIOGRID and STRING databases were used to search for the candidate proteins that may interact with HDGF and to predict the interaction between HDGF and C-JUN protein. The HDGF and C-JUN interaction in OC cells was detected by the endogenous Co-IP assay. Immunofluorescence analysis also showed that HDGF and C-JUN proteins were co-localized in the cytoplasm and nucleus. C-JUN is the key downstream transcription factor in the Wnt/β-catenin signaling pathway [9]. Studies have shown that C-JUN is an oncogenic transformation factor in OC. By activation of C-JUN-WEE1 signaling, diacylglycerol kinase alpha (DGKA) a metabolic kinase can promote platinum resistance in OC [27]. C-JUN can also regulate cell proliferation, migration, and metastasis by expression of various genes, including CCND1 [28–30]. CCND1 gene overexpression will affect the normal cell cycle. The main function of this protein is to regulate the cell cycle from the G1 stage of DNA synthesis to the S stage of DNA synthesis. Therefore, the key cell cycle proteins associated with the G1 to S phase transition were analyzed. We observed significant downregulation of C-JUN and CCND1 after NAP1L1 knockdown in OC cells. This result demonstrated that HDGF recruits C-JUN to participate in OC carcinogenesis. In complementary experiments, these effects were reversed by HDGF overexpression, including the increase in C-JUN and CCND1 expression and entry into the G1/S phase cell cycle. This result demonstrates that HDGF can recruit C-JUN to upregulate the expression of CCND1, thereby reversing the inhibitory effect of siNAP1L1 on OC cell proliferation.

## Conclusions

Taken together, our study indicates that NAP1L1 is a potential prognostic biomarker in OC and it can interact with HDGF to mediate the proliferation of OC cells. This process of triggered proliferation may contribute to activation of HDGF/C-JUN signaling in OC cells. We provide new insights into the role of NAP1L1 in OC progression.

## Supplementary Information

**Additional file 1:** **Table S1.**Transient and stable disturbance sequences. **Table S2.** The primers used in this study. **Table S3. **A list of Antibodies used for WB, Co-IP, IF and IHC.

**Additional file 2:** **Supplementary Figure 1. **The single protein function partner network of HDGF and C-JUN in BIOGRID (A) and STRING analysis(B).

**Additional file 3:** **Supplementary Figure 2. **NAP1L1 mRNA levels prognostic in The Kaplan-Meier Plotter data (*P* =0.013).

**Additional file 4:** **Supplementary Figure 3. **(A). Knocking down NAP1L1 reduced HDGF expression, and after the overexpression of NAP1L1 in SKOV3 and OVCAR3 cells (B), western blotting demonstrated that the protein level of HDGF is increased.

**Additional file 5.** Mass spectrometry assays raw data F016121

**Additional file 6.** Mass spectrometry assays raw data F016122

**Additional file 7.** The original Western blot image

## Data Availability

The datasets used and/or analyze in the current study are available from the corresponding author upon reasonable request.

## Abbreviations

CI: Confidence interval
HR: Hazard ratio
IHC: Immunohistochemistry
NAP1L1: Nucleosome assembly protein 1-like 1
SD: Standard deviation
SI: Staining index
HDGF: Heptoma-derived growth factor
C-JUN: Jun proto-oncogene
CCND1: Cyclin D1
OC: Ovarian cancer
CCK-8: Cell Counting Kit-8
Co-IP: Co-immunoprecipitation
TMA: Tissue microarrays
DFS: Disease free survival
OS: Overall survival rates
