# Supplementary material for: NAP1L1 promotes tumor proliferation through HDGF/C-JUN signaling in ovarian cancer
Source: BMC Cancer. 2022 Mar 29;22:339. doi: 10.1186/s12885-022-09356-z (PMC8962469; doi:10.1186/s12885-022-09356-z)
Supplement: Supplementary file 1 — Additional file 1: Table S1.Transient and stable disturbance sequences. Table S2. The primers used in this study. Table S3. A list of Antibodies used for WB, Co-IP, IF and IHC. [file 12885_2022_9356_MOESM1_ESM.docx]

**Additional Files**

**Supplementary Table：**

| TableS1.Transient and stable disturbance sequences | | | |
| --- | --- | --- | --- |
| **Gene** | **Accession** | **NO.** | **Target Seq** |
| NAP1L1 | Sh-NAP1L1 | Sh-NAP1L1 | GACAGTTCGTACTGTGACT |
| NAP1L1 | Si-NAP1L1 | Si-RNA1 | GCCAAGATTGAAGATGAGAAA |
|  | Si-NAP1L1 | Si-RNA2 | TTCCAATGACTCTTTCTTTAA |

| Table S2. The primers used in this study | | |
| --- | --- | --- |
| Primers name |  | Sequence(5’-3’) |
| NAP1L1 | Forward | TTTGCCCCTCCTGAAGTTCC |
|  | Reverse | CCCAACACAACTTGAGACATCC |
| HDGF | Forward | ATCAACAGCCAACAAATACC |
|  | Reverse | TTCTTATCACCGTCACCCT |
| c-JUN | Forward | TCAGACAGTGCCCGAGATG |
|  | Reverse | CTGCTGCGTTAGCATGAGTT |
| GAPDH | Forward | CATGGGTGTGAACCATGAGA |
|  | Reverse | GTCTTCTGGGTGGCAGTGAT |

| Table S3.A list of Antibodies used for WB, Co-IP, IF and IHC. | | | | |
| --- | --- | --- | --- | --- |
| antibodies | Cat. No | Company | Species | Dulution |
| NAP1L1 | mAb ab178687 | Abcam | Rabbit | 1:5000(WB); 1:200(IF); 1:20(Co-IP) |
| NAP1L1 | pAb#14898-1-AP | Proteintech | Rabbit | 1:500(WB); 1:500(IHC) |
| HDGF | mAb#60064-1-Ig | Proteintech | Mouse | 1:1000(WB); 1:50(IF); 1:10(Co-IP) |
| HDGF | pAb 11344-1-AP | Proteintech | Rabbit | 1:1000(WB) |
| c-JUN | mAb #9165 | Cell Sigaling | Rabbit | 1:1000(WB); 1:50(IF); 1:10(Co-IP) |
| CCND1 | mAb#6086-1-Ig | Proteintech | Mouse | 1:1000(WB) |
| GAPDH | pAb AP0063 | Bioworld | Rabbit | 1:10000(WB) |
