# Supplementary figures and images for: NAP1L1 promotes tumor proliferation through HDGF/C-JUN signaling in ovarian cancer
Source: BMC Cancer. 2022 Mar 29;22:339. doi: 10.1186/s12885-022-09356-z (PMC8962469; doi:10.1186/s12885-022-09356-z)

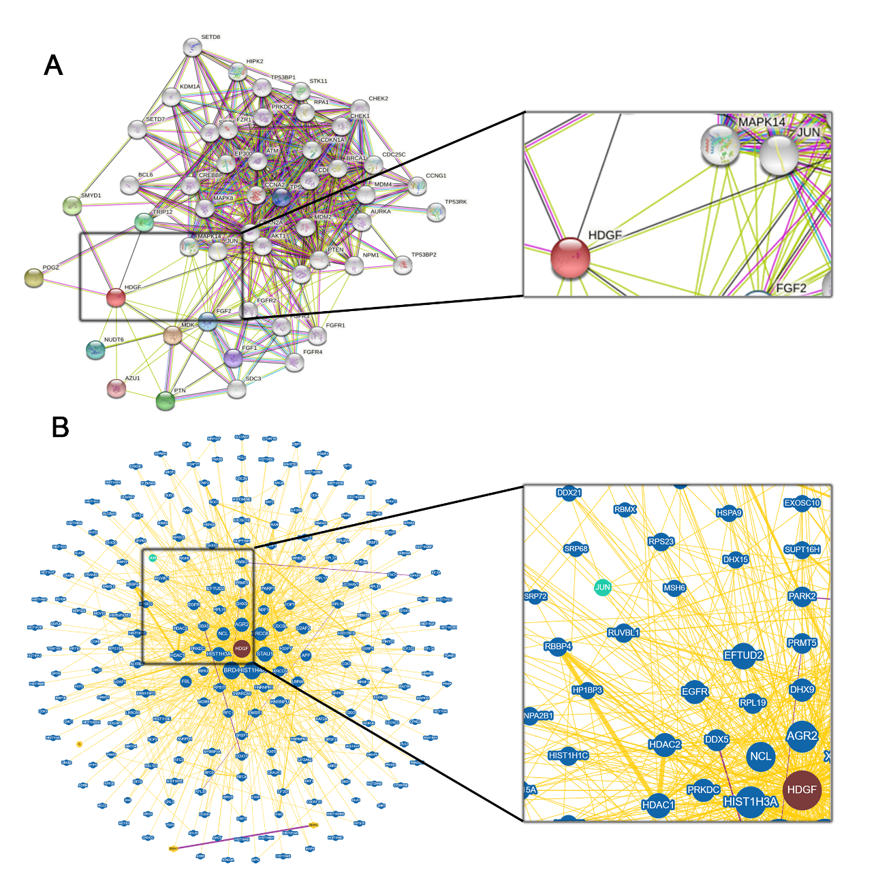

Supplement: Supplementary file 2 — Additional file 2: Supplementary Figure 1. The single protein function partner network of HDGF and C-JUN in BIOGRID (A) and STRING analysis(B). [file 12885_2022_9356_MOESM2_ESM.tif]

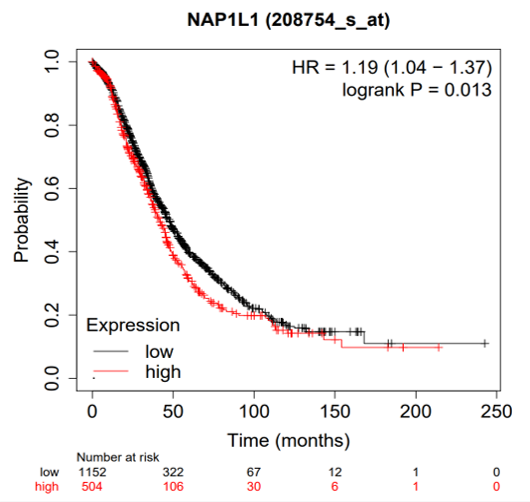

Supplement: Supplementary file 3 — Additional file 3: Supplementary Figure 2. NAP1L1 mRNA levels prognostic in The Kaplan-Meier Plotter data (P =0.013). [file 12885_2022_9356_MOESM3_ESM.tif]

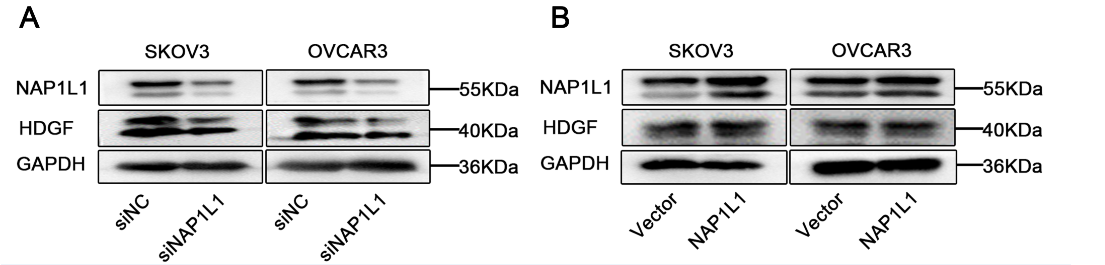

Supplement: Supplementary file 4 — Additional file 4: Supplementary Figure 3. (A). Knocking down NAP1L1 reduced HDGF expression, and after the overexpression of NAP1L1 in SKOV3 and OVCAR3 cells (B), western blotting demonstrated that the protein level of HDGF is increased. [file 12885_2022_9356_MOESM4_ESM.tif]

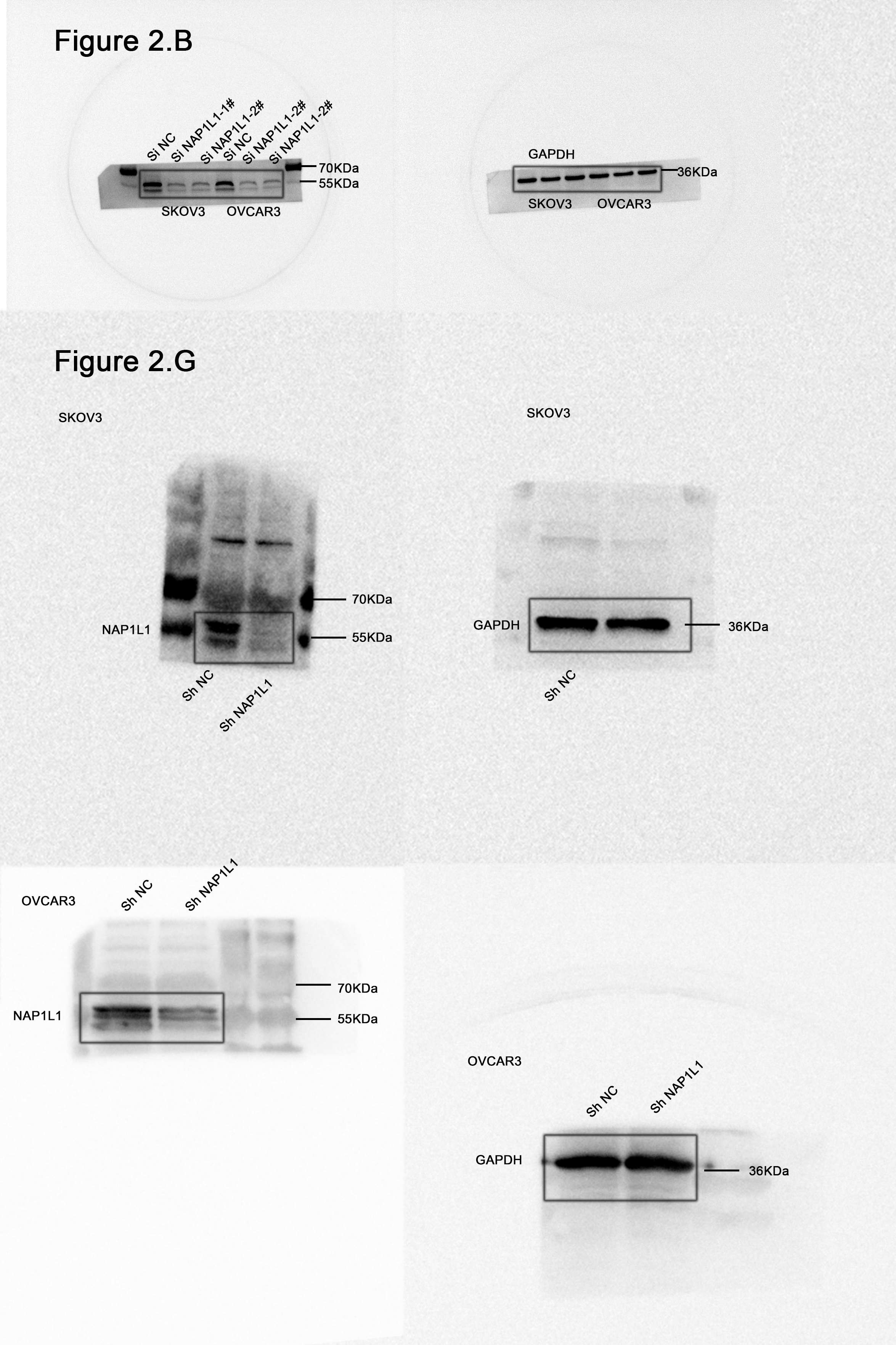


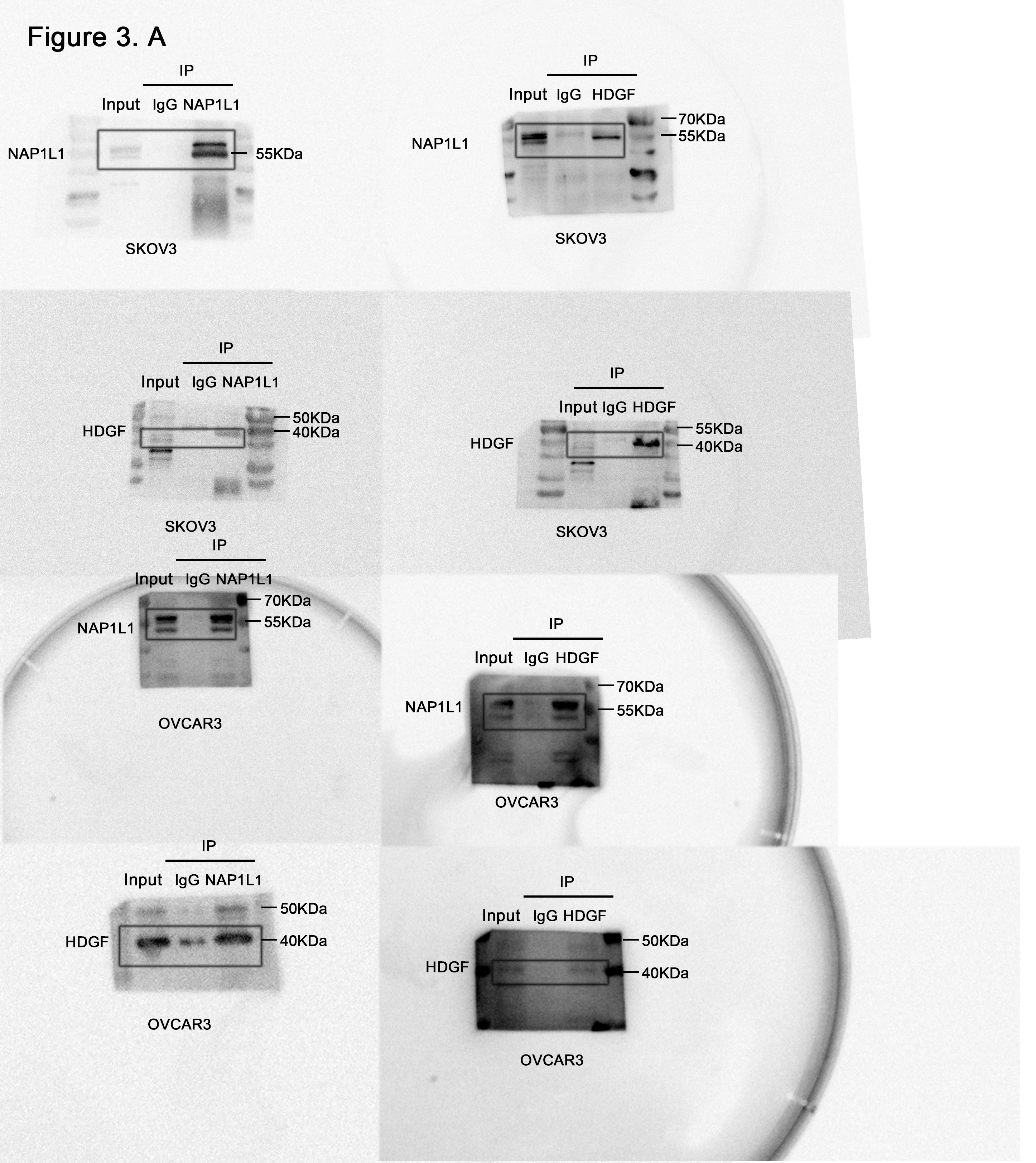


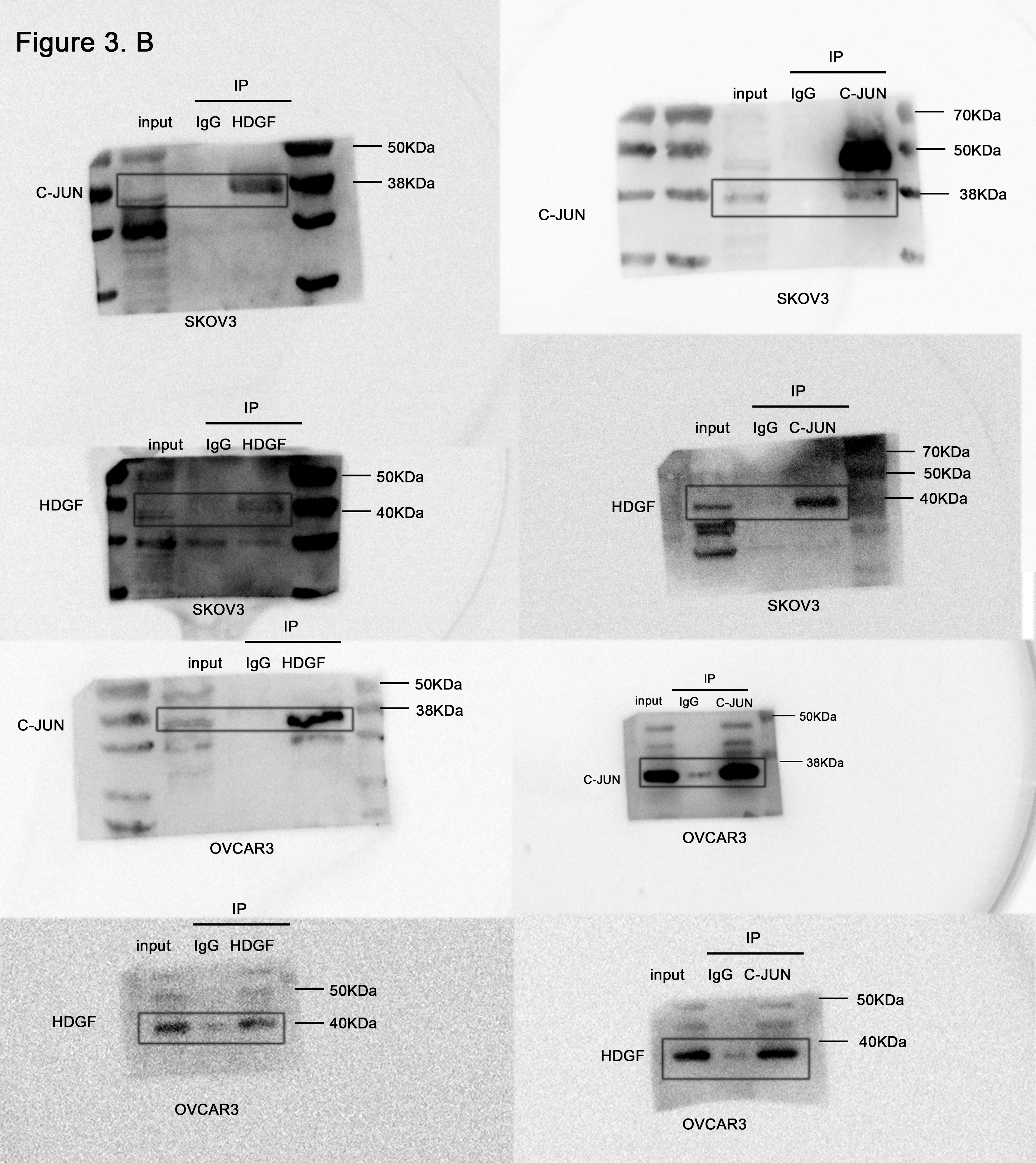


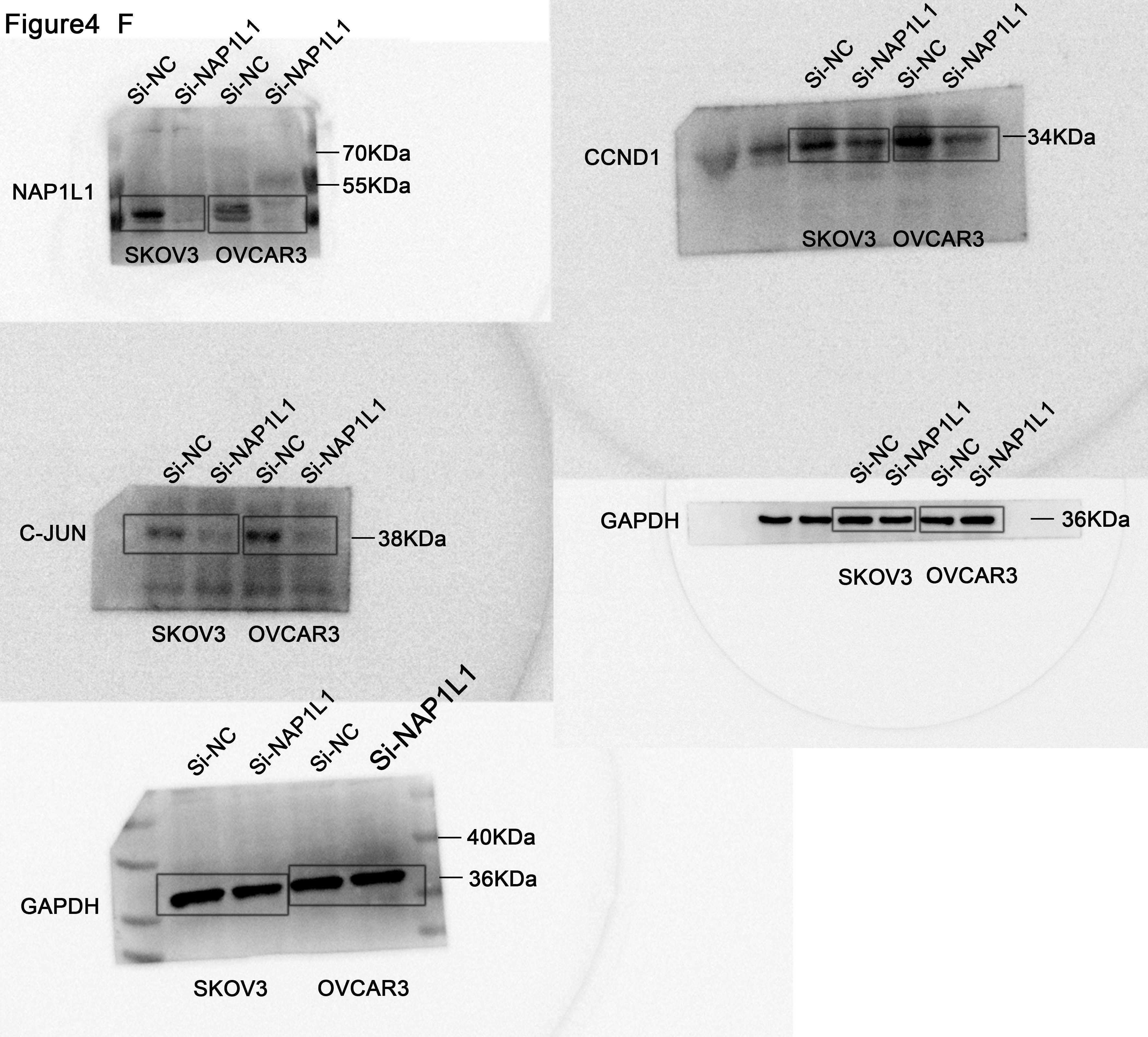


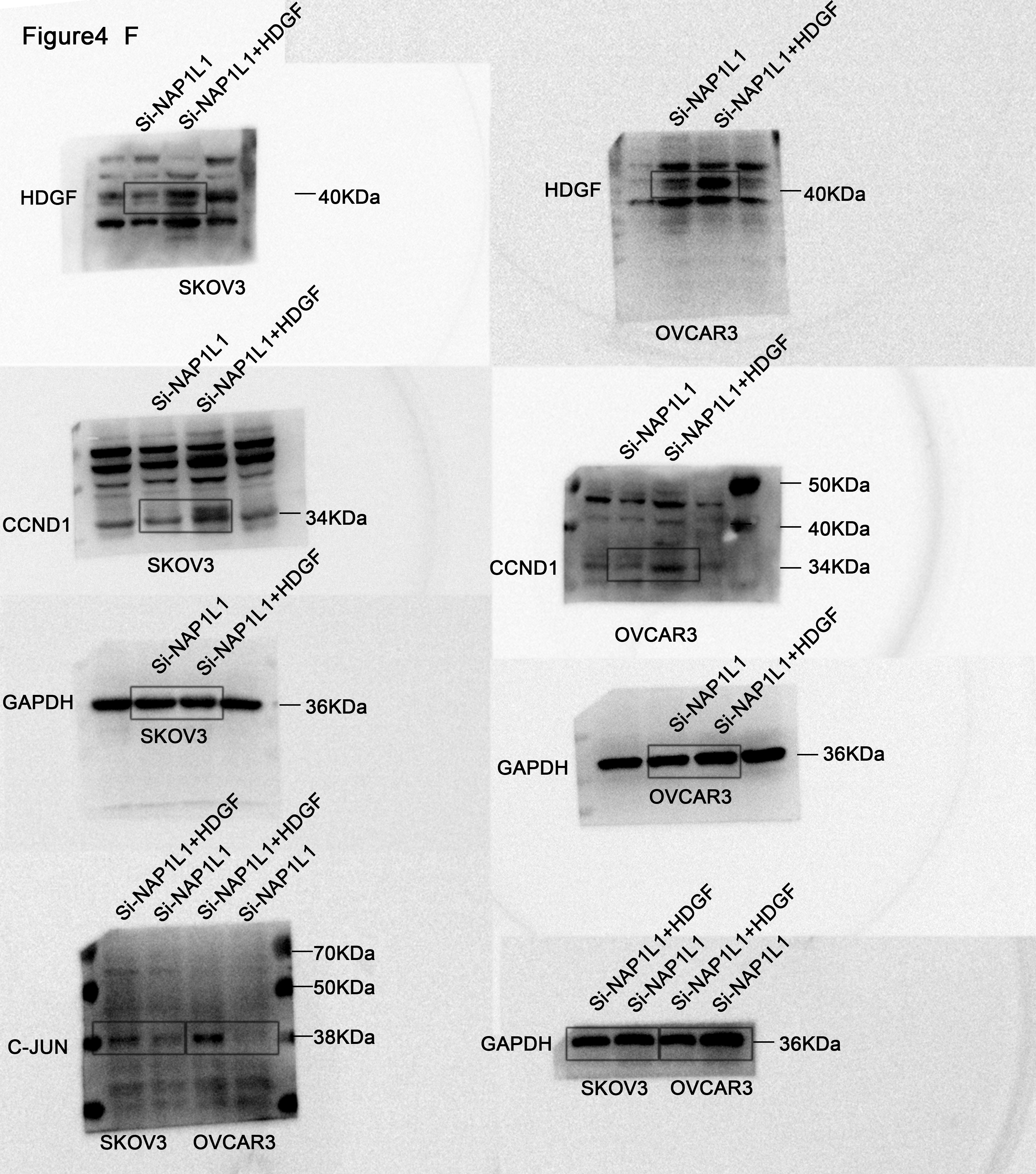

Supplement: Supplementary file 7 — Additional file 7. The original Western blot image [file 12885_2022_9356_MOESM7_ESM.docx]
